# Supplementary material for: Duration and key determinants of infectious virus shedding in hospitalized patients with coronavirus disease-2019 (COVID-19)
Source: Nat Commun. 2021 Jan 11;12:267. doi: 10.1038/s41467-020-20568-4 (PMC7801729; doi:10.1038/s41467-020-20568-4)
Supplement: Supplementary file 2 — Supplementary Information [file 41467_2020_20568_MOESM2_ESM.pdf]

**Supplementary information: Duration and key determinants of infectious virus shedding in hospitalized patients with coronavirus disease-2019 (COVID-19)**

**Supplementary Figure 1.** Qualitative assessment of sgRNA in lower respiratory tract samples.

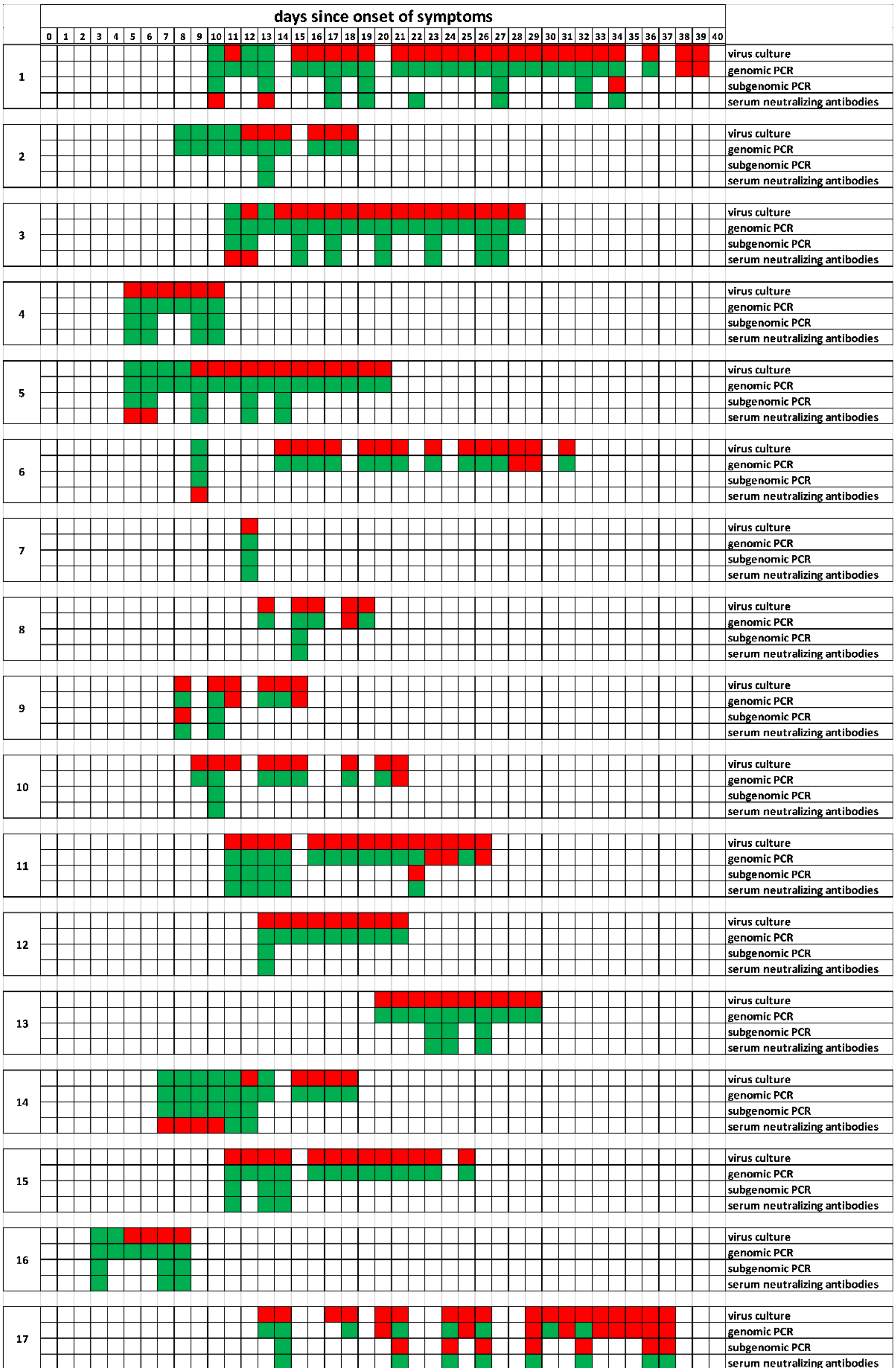


Individual patient charts with virological test results (positive/negative) in relation to the duration of symptoms for patients for whom sgRNA RT-PCR results of lower respiratory tract samples were available. Positive test results are depicted in green and negative test results in red.

**Supplementary Figure 2.** Qualitative assessment of sgRNA in upper respiratory tract samples.

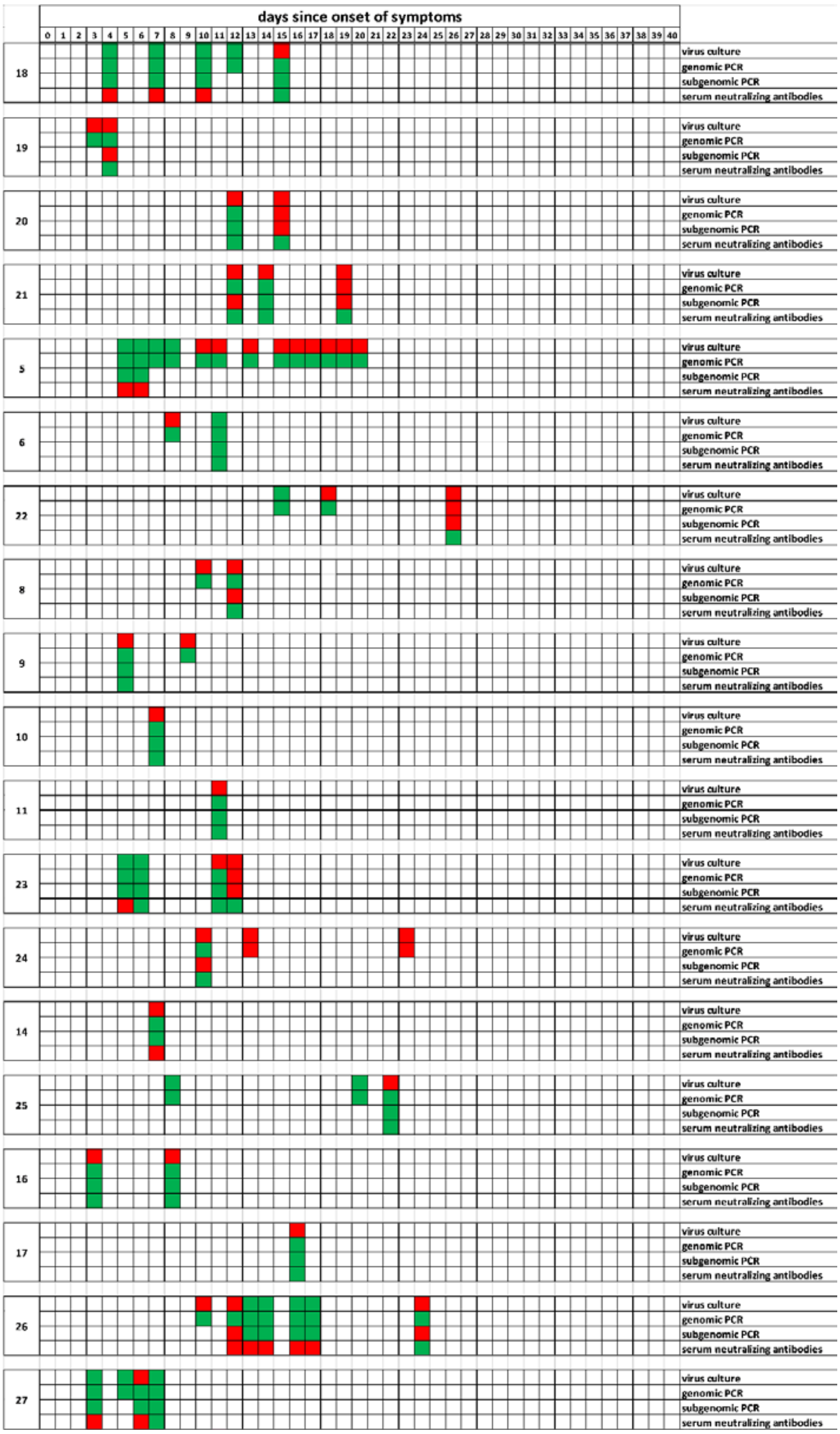


Individual patient charts with virological test results (positive/negative) in relation to the duration of symptoms for patients for whom sgRNA RT-PCR results of upper respiratory tract samples were available. Positive test results are depicted in green and negative test results in red.

**Supplementary Figure 3.** Quantitative assessment of sgRNA in respiratory tract samples.

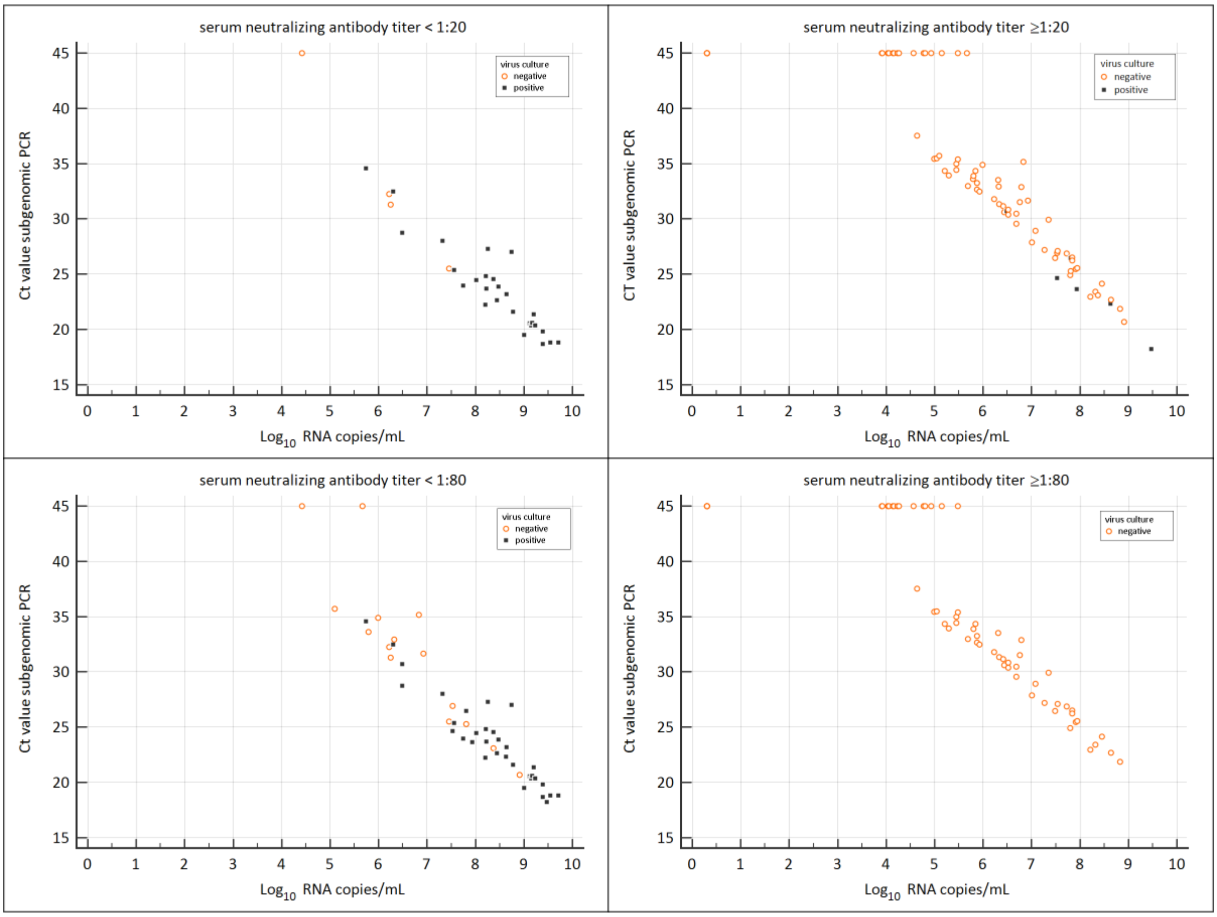


Plots of cycle threshold value of the viral subgenomic RNA RT-PCR (y-axis) versus the genomic viral load (x-axis) for patients with an undetectable neutralizing antibody response (upper left panel), a detectable neutralizing antibody response (upper right panel), neutralizing antibody titers < 1:80 (lower left panel), and neutralizing antibody titers ≥ 1:80. Cycle threshold values are inversely correlated to the subgenomic RNA load and a cycle threshold > 45 is regarded as a negative test result. Ct values of the subgenomic RNA correlated well with genomic viral RNA load, but had no added value over this measure or antibody response to predict a positive virus culture.

**Supplementary Table 1 – Results of the univariate sensitivity analysis**

| Characteristic | Culture positive (n=33) | Culture negative (n=79) | Odds ratio (95% confidence interval) | p | QIC |
|---|---|---|---|---|---|
| Viral load (RNA copies/mL) | | | | | |
| > 7 $log_{10}$ | 29 (88%) | 22 (29%) | 18.8 (5.5- 64.2) | <0.001 | 105.3 |
| > 8 $log_{10}$ | 23 (70%) | 7 (9%) | 23.7 (9.3- 60.4) | <0.001 | 98.3 |
| Duration of illness | | | | | |
| < 7 days | 12 (36%) | 6 (8%) | 7.0 (1.5-32.7) | 0.01 | 131.3 |
| <10 days | 20 (61%) | 17 (22%) | 5.6 (1.7-18.1) | 0.004 | 128.0 |
| <14 days | 30 (91%) | 41 (52%) | 9.3 (1.1-80.3) | 0.04 | 128.3 |
| PRNT response | | | | | |
| Detectable ≥1:20 | 6 (18%) | 75 (95%) | 0.01 (0.003-0.05) | <0.001 | 69.9 |
| Immunosuppression | | | | | |
| Mild/moderate | 10 (30%) | 10 (13%) | 3.0 (0.8-11.0) | 0.098 | 139.1 |
| Severe | 4 (12%) | 6 (18%) | 1.7 (0.3-9.7) | 0.57 | 143.5 |
| Viral load and PRNT response * | | | | | |
| Viral load > 7 $log_{10}$ & PRNT ≥1:20 | 5 (15%) | 21 (27%) | 0.54 (0.01 – 23.3) | 0.75 | 59.6 |

| | | | | | |
|---|---|---|---|---|---|
| Viral load > 7 $log_{10}$ | | | 24.0 (2.0 – 281.9) | 0.011 | |
| PRNT ≥ 1:20 | | | 0.02 (0.001 – 0.34) | 0.007 | |

***** The interaction between viral load and PRNT response (defined as ≥1:20) was investigated to determine a possible effect modification between the two variables. The numbers for the interaction between a viral load >8 $log_{10}$ per ml and a PRNT response were too low resulting in infinite upper limits of the 95% confidence intervals. Generalized estimating equations were used to identify factors that are associated with a virus culture positive respiratory tract sample. The continuous data in the generalized estimating equations were dichotomized using various cut-off values. In Table S1, we present the results of the univariate sensitivity analysis in which we show that choosing a different cut-off value for dichotomizing the different dependent variables did not have an impact on which of these variables had a statistically significant and independent impact on a positive culture and therefore of finding an infectious virus. For each of the cut-offs we also calculated the quasi-likelihood under the independence model criterion (QIC) as a criterion to identify the best fitting generalized estimating equation model. The statistical model with the lowest QIC values, indicating the best fit, has been presented in the main paper (see Table 3).

**Supplementary Table 2 – sensitivity analysis of the multivariate analysis**

| Characteristic | Cut-off | Odds ratio (95% confidence interval) | p | QIC |
|---|---|---|---|---|
| Viral load<br>Duration of illness<br>PRNT<br>Immunosuppression | > 7 $log_{10}$<br>< 7 days<br>Detectable (1:20)<br>yes | 14.7 (3.7-58.1)<br>2.1 (0.4-11.8)<br>0.01 (0.003-0.08)<br>2 (0.7-5.3) | <0.001<br>0.40<br><0.001<br>0.17 | 57.6 |
| Viral load<br>Duration of illness<br>PRNT<br>Immunosuppression | > 7 $log_{10}$<br>< 10 days<br>Detectable (1:20)<br>yes | 14.3 (3.1-65.6)<br>2.5 (0.5-11.7)<br>0.01 (0.002-0.09)<br>1.5 (0.5-4.9) | <0.001<br>0.26<br><0.001<br>0.49 | 61.5 |
| Viral load<br>Duration of illness<br>PRNT<br>Immunosuppression | > 7 $log_{10}$<br>< 14 days<br>Detectable (1:20)<br>yes | 14.8 (3.5-62.4)<br>2.3 (0.4-13.4)<br>0.01 (0.002-0.09)<br>1.6 (0.6-4.6) | <0.001<br>0.37<br><0.001<br>0.35 | 58.5 |
| Viral load<br>Duration of illness<br>PRNT<br>Immunosuppression | > 8 $log_{10}$<br>< 7 days<br>Detectable (1:20)<br>yes | 10.7 (3.7-30.8)<br>5 (0.6-42.6)<br>0.03 (0.006-0.13)<br>1.7 (0.5-5.8) | <0.001<br>0.14<br><0.001<br>0.40 | 61.4 |
| Viral load<br>Duration of illness<br>PRNT<br>Immunosuppression | > 8 $log_{10}$<br>< 10 days<br>Detectable (1:20)<br>yes | 8.6 (3-25)<br>3.1 (0.5-20.5)<br>0.02 (0.005-0.12)<br>1.2 (0.3-5.5) | <0.001<br>0.24<br><0.001<br>0.82 | 65.3 |
| Viral load<br>Duration of illness<br>PRNT<br>Immunosuppression | > 8 $log_{10}$<br>< 14 days<br>Detectable (1:20)<br>yes | 9.8 (3.7-26.0)<br>4.5 (0.7-28.0)<br>0.03 (0.006-0.12)<br>1.3 (0.4-4.0) | <0.001<br>0.11<br><0.001<br>0.70 | 63.4 |

Generalized estimating equations were used to identify factors that are associated with a virus culture positive respiratory tract sample. The continuous data in the generalized estimating equations were dichotomized using various cut-off values. In Table S2, we present the results of the multivariate sensitivity analysis in which we show that choosing a different cut-off value for dichotomizing the different dependent variables did not have an impact on which of these variables had a statistically significant and independent impact on a positive culture and therefore of finding

an infectious virus. For each of the cut-offs we also calculated the quasi-likelihood under the independence model criterion (QIC) as a criterion to identify the best fitting generalized estimating equation model. The statistical model with the lowest QIC values, indicating the best fit, has been presented in the main paper (see Table 3).
