# Supplementary material for: Duration and key determinants of infectious virus shedding in hospitalized patients with coronavirus disease-2019 (COVID-19)
Source: Nat Commun. 2021 Jan 11;12:267. doi: 10.1038/s41467-020-20568-4 (PMC7801729; doi:10.1038/s41467-020-20568-4)
Supplement: Supplementary file 3 — Reporting Summary [file 41467_2020_20568_MOESM3_ESM.pdf]

nature research

Corresponding author(s): Jeroen J.A. van Kampen

Last updated by author(s): Nov 25, 2020

Reporting Summary

Nature Research wishes to improve the reproducibility of the work that we publish. This form provides structure for consistency and transparency in reporting. For further information on Nature Research policies, see our Editorial Policies and the Editorial Policy Checklist.

Statistics

For all statistical analyses, confirm that the following items are present in the figure legend, table legend, main text, or Methods section.

n/a	Confirmed	
☐	☒	The exact sample size (*n*) for each experimental group/condition, given as a discrete number and unit of measurement
☐	☒	A statement on whether measurements were taken from distinct samples or whether the same sample was measured repeatedly
☐	☒	The statistical test(s) used AND whether they are one- or two-sided<br>*Only common tests should be described solely by name; describe more complex techniques in the Methods section.*
☐	☒	A description of all covariates tested
☐	☒	A description of any assumptions or corrections, such as tests of normality and adjustment for multiple comparisons
☐	☒	A full description of the statistical parameters including central tendency (e.g. means) or other basic estimates (e.g. regression coefficient) AND variation (e.g. standard deviation) or associated estimates of uncertainty (e.g. confidence intervals)
☐	☒	For null hypothesis testing, the test statistic (e.g. *F*, *t*, *r*) with confidence intervals, effect sizes, degrees of freedom and *P* value noted<br>*Give P values as exact values whenever suitable.*
☒	☐	For Bayesian analysis, information on the choice of priors and Markov chain Monte Carlo settings
☒	☐	For hierarchical and complex designs, identification of the appropriate level for tests and full reporting of outcomes
☒	☐	Estimates of effect sizes (e.g. Cohen's *d*, Pearson's *r*), indicating how they were calculated

Our web collection on statistics for biologists contains articles on many of the points above.

Software and code

Policy information about availability of computer code

Data collection	LabTrain version 3 (bodegro, Netherlands), HiX version 6.1 (Chipsoft, Netherlands), Excel 2016 (Microsoft Corp, USA).
Data analysis	geepack package version 1.3-1 and R version 4.0.0. (R Foundation), MedCalc version 19.2.3. (MedCalc Software Ltd).

For manuscripts utilizing custom algorithms or software that are central to the research but not yet described in published literature, software must be made available to editors and reviewers. We strongly encourage code deposition in a community repository (e.g. GitHub). See the Nature Research guidelines for submitting code & software for further information.

Data

Policy information about availability of data

All manuscripts must include a data availability statement. This statement should provide the following information, where applicable:

- Accession codes, unique identifiers, or web links for publicly available datasets
- A list of figures that have associated raw data
- A description of any restrictions on data availability

Figure 1 and 2, and supplementary Figure 1 and 2 have associated raw data.
Source data are provided with this manuscript. All further relevant data are available from the authors upon request.

Field-specific reporting

Please select the one below that is the best fit for your research. If you are not sure, read the appropriate sections before making your selection.

- [x] Life sciences
- [ ] Behavioural & social sciences
- [ ] Ecological, evolutionary & environmental sciences

For a reference copy of the document with all sections, see nature.com/documents/nr-reporting-summary-flat.pdf

Life sciences study design

All studies must disclose on these points even when the disclosure is negative.

Sample size	The sample size was based on availability of the samples and patients.
Data exclusions	No data were excluded from the analyses.
Replication	All PRNT50 measurements and virus cultures were peformed in duplo. All replicates were successful. Molecular tests (sgRNA and genomic RNA) were performed once. Virus cultures and genomic RNA PCRs were performed in an ISO15189:2012 accredited setting.
Randomization	Not relevant for this study, because all patient samples and data used in this study were collected in the context of routine clinical patient care.
Blinding	No formal blinding was performed in this study. However, the technicians who performed the assays do not have access to the electronic patients files, i.e. technicians don't have access to clinical data.

Reporting for specific materials, systems and methods

We require information from authors about some types of materials, experimental systems and methods used in many studies. Here, indicate whether each material, system or method listed is relevant to your study. If you are not sure if a list item applies to your research, read the appropriate section before selecting a response.

Materials & experimental systems

n/a	Involved in the study	
[ ]	[x]	Antibodies
[ ]	[x]	Eukaryotic cell lines
[x]	[ ]	Palaeontology and archaeology
[x]	[ ]	Animals and other organisms
[x]	[ ]	Human research participants
[ ]	[x]	Clinical data
[x]	[ ]	Dual use research of concern

Methods

n/a	Involved in the study	
[x]	[ ]	ChIP-seq
[x]	[ ]	Flow cytometry
[x]	[ ]	MRI-based neuroimaging

Antibodies

Antibodies used	polyclonal rabbit SARS-CoV anti-nucleoprotein antibodies (Sino Biological, catalogue number 40143-T62) and Alexa Fluor 488 - labeled polyclonal goat anti-rabbit IgG (Invitrogen, catalogue number A-11070)
Validation	Okba NMA, Müller MA, Li W, Wang C, GeurtsvanKessel CH, Corman VM, Lamers MM, Sikkema RS, de Bruin E, Chandler FD, Yazdanpanah Y, Le Hingrat Q, Descamps D, Houhou-Fidouh N, Reusken CBEM, Bosch BJ, Drosten C, Koopmans MPG, Haagmans BL. Severe Acute Respiratory Syndrome Coronavirus 2-Specific Antibody Responses in Coronavirus Disease Patients. Emerg Infect Dis. 2020 Jul;26(7):1478-1488. doi: 10.3201/eid2607.200841.

Eukaryotic cell lines

Policy information about cell lines

Cell line source(s)	Vero cells: Kuiken T, van den Hoogen BG, van Riel DA, Laman JD, van Amerongen G, Sprong L, Fouchier RA, Osterhaus AD. Experimental human metapneumovirus infection of cynomolgus macaques (Macaca fascicularis) results in virus replication in ciliated epithelial cells and pneumocytes with associated lesions throughout the respiratory tract. Am J Pathol. 2004 Jun;164 (6):1893-900. doi: 10.1016/S0002-9440(10)63750-9.
Authentication	cell line was not authenticated for this study.
Mycoplasma contamination	All cell lines used in this study tested negative for Mycoplasma species by PCR.

Commonly misidentified lines (See ICLAC register)	None

Clinical data

Policy information about clinical studies

All manuscripts should comply with the ICMJEguidelines for publication of clinical research and a completedCONSORT checklist must be included with all submissions.

Clinical trial registration	None.
Study protocol	Not available. This is not a clinical trial.
Data collection	All patient samples and data used in this study were collected in the ocntact of routine clinical patient care. Additional analyses were performed inly on surplus of patient material collected in the context of routine patient care.
Outcomes	None.
